# Assessment of Effectiveness of the Algorithm for Automated Quantitative Analysis of Metallic Strut Tissue Short-Term Coverage with Intravascular Optical Coherence Tomography

**DOI:** 10.3390/jcm13154336

**Published:** 2024-07-25

**Authors:** Joanna Fluder-Wlodarczyk, Zofia Schneider, Tomasz Pawłowski, Wojciech Wojakowski, Pawel Gasior, Elżbieta Pociask

**Affiliations:** 1 Division of Cardiology and Structural Heart Diseases, Medical University of Silesia in Katowice, 40-635 Katowice, Polandp.m.gasior@gmail.com (P.G.); 2 Faculty of Geology, Geophysics and Environmental Protection, AGH University of Kraków, 30-059 Krakow, Poland; 3 Department of Biocybernetics and Biomedical Engineering, AGH University of Kraków, 30-059 Kraków, Poland; elzbieta.pociask@gmail.com

**Keywords:** coronary artery disease, optical coherence tomography, OCT, intravascular imaging, PCI

## Abstract

**Background:** Due to its high resolution, optical coherence tomography (OCT) is the most suitable modality for neointimal coverage assessments. Evaluation of stent healing seems crucial to accurately define their safety profile since delayed healing is connected with stent thrombosis. This study aimed to present an algorithm for automated quantitative analysis of stent strut coverage at the early stages of vessel healing in intravascular OCT. **Methods:** A set of 592 OCT frames from 24 patients one month following drug-eluting stent implantation was used to assess the algorithm’s effectiveness. Struts not covered on any side or covered but only on one side were categorized as uncovered. The algorithm consists of several key steps: preprocessing, vessel lumen segmentation, automatic strut detection, and measurement of neointimal thickness. **Results:** The proposed algorithm proved its efficiency in lumen and stent area estimation versus manual reference. It showed a high positive predictive value (PPV) (89.7%) and true positive rate (TPR) (91.4%) in detecting struts. A qualitative assessment for covered and uncovered struts was characterized by high TPR (99.1% and 80%, respectively, for uncovered and covered struts) and PPV (77.3% and 87%). **Conclusions:** The proposed algorithm demonstrated good agreement with manual measurements. Automating the stent coverage assessment might facilitate imaging analysis, which might be beneficial in experimental and clinical settings.

## 1. Introduction

Intravascular optical coherence tomography (OCT) provides detailed in vivo visualization of native coronary arteries and implanted stents, which translates into favorable clinical outcomes for patients treated with percutaneous coronary intervention (PCI) [1,2,3,4]. Due to high-resolution images, OCT enables the detection and quantification of the tissue covering the stent surface [5], providing insights into the stent healing process incomparable to other intravascular imaging modalities [6,7,8]. Also, it is worth emphasizing that several studies have revealed good agreement between OCT and histological analysis of neointimal coverage [9,10,11].

PCI with stent deployment is followed by mandatory dual antiplatelet therapy (DAPT) to protect patients from stent thrombosis (ST) [12]. DAPT protects against ischemic complications; however, especially prolonged DAPT increases the risk of bleeding [13]. This is essential, since around 40% of patients undergoing PCI are considered as high bleeding risk patients (HBR) [14,15]. Data suggest that HBR patients have a higher prevalence of both bleeding and ischemic events. This population does not benefit from prolonged DAPT even after complex PCI or acute coronary syndrome (ACS) [16]. Furthermore, in the general population, post-discharge bleeding is not unusual and is strongly associated with cardiac and all-cause mortality regardless of whether a transfusion was needed or not [17]. Therefore, the duration of DAPT must be carefully considered to balance ischemic and bleeding complications.

Since delayed healing is the most significant risk factor for ST [18,19,20], early strut coverage after PCI seems crucial to achieving better clinical outcomes. Therefore, OCT is the most suitable in vivo modality for evaluating the safety of various stent platforms. However, manual analysis of neointimal coverage is tedious and time-consuming. This flaw makes assessment of stent healing impractical in daily practice, which the automatization of this process might overcome. Therefore, we present an algorithm for automated quantitative analysis of stent strut coverage at the early stages of vessel healing in intravascular OCT.

## 2. Materials and Methods

### 2.1. Algorithm

The algorithm was developed by engineers from the AGH University of Krakow. The algorithm includes the following steps: preprocessing (image enhancement and artifact removal), lumen segmentation, stent strut shadow detection and segmentation, feature extraction, and strut classification. The proposed algorithm processes each frame from the entire OCT image dataset. The multi-frame images are saved in DICOM format, and each frame is a 2D RGB image in a Cartesian coordinate system. Preprocessing begins with the removal of all markers and text notes using a color pixel mask. A polar transformation is then applied, converting the circular shape of the coronary artery in cross-sectional view into an upright structure. This transformation uses a polar coordinate system in which each point on the plane is defined by its distance from a reference point and its angle from a reference direction.

The next step, the segmentation of the vessel lumen, involves an algorithm based on binarization and an active contour model [21]. The method employs a binarization technique to convert the grayscale OCT image into a binary image. This process involves selecting a threshold value to differentiate between the lumen (vessel interior) and the surrounding tissues. Pixels with intensity values above the threshold are marked as lumen, while those below are marked as non-lumen. After binarization, an active contour model, also known as a “snake”, is applied. This model iteratively evolves to fit the lumen boundary by minimizing an energy function that considers both image gradients and the contour’s smoothness. The active contour model helps to refine the initial segmentation obtained from binarization by accurately delineating the lumen boundary. Finally, post-processing steps are applied to further refine the segmented lumen boundary. These steps include morphological operations to smooth the contour and remove small segmentation errors or noise.

The defined vessel lumen boundary is then used to create a ring-shaped mask covering the vessel wall tissue. The thickness of the mask is determined adaptively based on the pixel intensity distribution. Adaptive thresholding is then carried out on the red component of the image within the mask to segment the shadows of the stent struts. To remove destructive speckle effects without damaging the borders, a median filter and Gaussian smoothing operator are applied to reduce unnecessary detail and background noise. A series of morphological operations, including opening and closing, are performed to minimize the impact of artifacts on the final result. Opening and closing preserve the area of interest, as opposed to erosion and dilation, which can change its size.

Image intensity profiles passing through each object are then determined. On intravascular OCT images, the metal stent strut appears as a bright spot with a trailing shadow, reflecting most of the light, while normal vessel tissue scatters and attenuates the light. Therefore, the strut has higher intensity values than the surrounding tissue. The pixels with the maximum intensity value in each A-line are candidates, assuming only one strut per A-line, excluding overlapping stents. Struts do not always have the highest intensity values throughout the pullback run, making a global intensity threshold impractical. Instead, the slope of the intensity profile is used. Each profile undergoes analysis. After satisfying specific conditions, such as a peak exceeding the height threshold followed by a clear drop in intensity, the object is considered a shadow of the stent strut, and the peak in the profile is recognized as part of the strut. The position of the stent strut is defined by the midpoint of its anterior edge.

**Figure 1:**
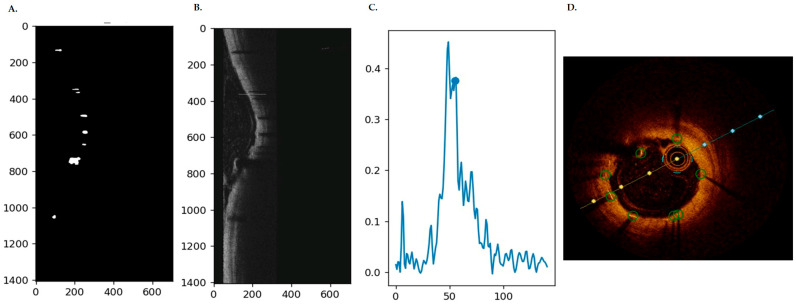
(**A**) Identification of candidates, i.e., areas containing potential stent struts—segmentation of strut shadows in polar coordinates (white objects). For each object, image intensity profiles were generated (**B**,**C**) and, based on these profiles, features were extracted that helped distinguish stent struts from abnormally segmented areas and finally marked on the original images in Cartesian coordinates (**D**).

After all operations, the resulting polar image is transformed into an image in Cartesian coordinates. The thickness of the neointima is then measured as the distance between the lumen and the stent contour. Figure 1 graphically presents how the proposed algorithm works.

### 2.2. Materials

The algorithm was validated using 592 frames from 24 pullbacks from 24 patients that underwent OCT imaging follow-up one month following OCT-guided DES implantation in the Division of Cardiology and Structural Heart Diseases, Medical University of Silesia in Katowice. The OCT procedure was performed on average 32 (±3) days after stent deployment in a coronary artery (left anterior descending (LAD), *n* = 7; left circumflex (LCx), *n* = 11; and right coronary (RCA), *n* = 6). Out of the 24 patients, 10 received bioabsorbable polymer-coated sirolimus-eluting stent Alex Plus (BP-SES, Alex Plus, Balton, Warsaw, Poland), while the remaining 14 patients received durable-polymer zotarolimus-eluting stent Onyx (ZES, Resolute Onyx, Medtronic, Minneapolis, MN, USA). All patients gave written informed consent. OCT imaging one month following DES implantation was approved by the local bioethical committee. The OCT examinations were performed in 2017–2019 using the ILUMIEN OPTIS system and Dragonfly catheters (Abbott, Green Oaks, IL, USA). During the OCT imaging, an automated pullback triggered by contrast injection was used. All patients were treated with unfractionated heparin prior to the OCT procedure to obtain an activated clotting time (ACT) of >250 s.

Experienced analysts carried out manual measurements using the CAAS Intravascular 2.0 software (Pie Medical, Maastricht, The Netherlands). Before analyzing the data, all patient information was anonymized. The measurements included lumen area, stent area, neointimal thickness and each strut was classified as either covered or uncovered. Struts not covered on any side or covered but only on one side were categorized as uncovered.

### 2.3. Statistical Analysis

Statistical analysis was performed using R software (R version 4.3.2 (31 October 2023) R: A language and environment for statistical computing. R Foundation for Statistical Computing, Vienna, Austria). Categorical data are presented as absolute numbers and percentages. Continuous variables were given as mean (SD) or median (IQR). The distribution of continuous variables was compared using the Mann–Whitney–Wilcoxon test. 

The threshold for statistical significance was *p* < 0.05. Stent detection and classification were validated in terms of both positive predictive value (PPV; precision) and true-positive rate (TPR; sensitivity). These were calculated as follows:

$$PPV=\frac{NUMBER\;OF\;TRUE\;POSITIVES}{NUMBER\;OF\;TRUE\;POSITIVES\;+\;NUMBER\;OF\;FALSE\;POSITIVES}$$

$$TPR=\frac{NUMBER\;OF\;TRUE\;POSITIVES}{NUMBER\;OF\;TRUE\;POSITIVES\;+\;NUMBER\;OF\;FALSE\;NEGATIVES}$$

The agreement between the algorithm and manual measurements was assessed using Bland–Altman plots.

## 3. Results

**Figure 2:**
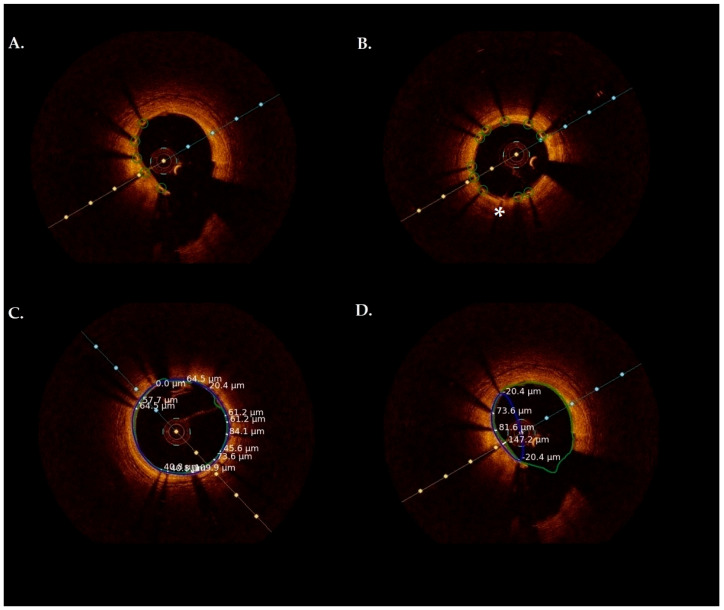
Sample results of frames analyzed by the proposed algorithm. (**A**) Successful struts detection (green circles). (**B**) Partially failed strut detection, one strut is missed by the algorithm (asterisk). (**C**) Sample frame with measurements of neointimal thickness, marked lumen area (green) and stent area (blue). (**D**) Failed stent area detection (blue), because visible struts are located on a small part of the stent circumference.

**Figure 3:**
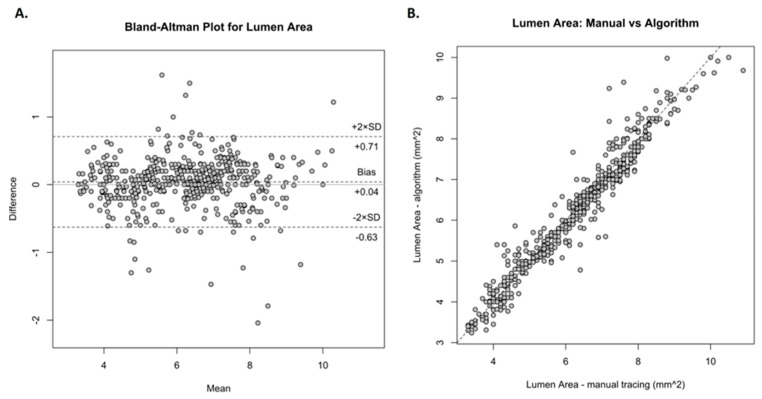
Comparison of manually measured lumen area and by the algorithm. (**A**) Bland–Altman plot. Most of the results are within the range of two deviations, with mean differences +0.04 and the limit of agreement is −0.63 to +0.71. (**B**) Correlation plot demonstrating a positive correlation with a Pearson correlation coefficient R = 0.96.

**Figure 4:**
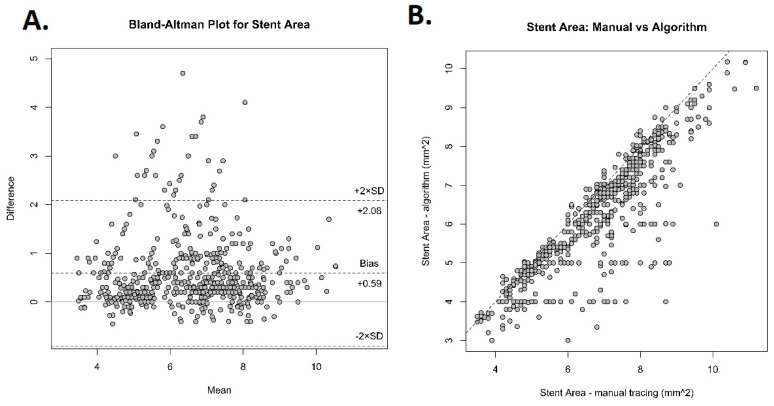
Comparison of manually measured stent area and by the algorithm. (**A**) Bland–Altman plot with mean differences +0.59, which indicates that the algorithm tends to underestimate stent area. (**B**) Correlation plot demonstrating a positive correlation with a Pearson correlation coefficient R = 0.85.

Figure 2 presents frames, which were analyzed by the algorithm. The first step involved determining the lumen area using the vessel lumen segmentation method. Figure 3 demonstrates correlation analysis and Bland–Altman statistics for lumen area estimated manually versus by the algorithm. The Bland–Altman plot reveals generally good agreement between both tested methods. The mean difference (mean difference +0.04) was near zero, indicating no bias in the automated measurements. Also, the correlation plot shows a strong, positive correlation with a Pearson correlation coefficient of R = 0.96. The agreement between manual analyses and the algorithm in measuring stent area is presented in Figure 4. The Bland–Altman plot shows that the algorithm slightly underestimated the stent area (mean difference +0.59). However, those two methods still had a positive correlation with a Pearson correlation coefficient of R = 0.85.

The detection and qualification of struts as covered or uncovered, were validated using two parameters—PPV and TPR. The analyst identified 5668 struts, while the algorithm detected 5763 struts. Among these, 1338 and 1719 were classified as uncovered, and 4330 and 3467 as covered, respectively. The detection of struts was characterized by good PPV (89.7%) and TPR (91.4%). Identifying uncovered struts was associated with better TPR (99.1%) but lower PPV (77.3%), while covered struts were recognized with higher PPV (87.0%) but lower TPR (80.0%). These findings are summarized in Table 1.

**Figure 5:**
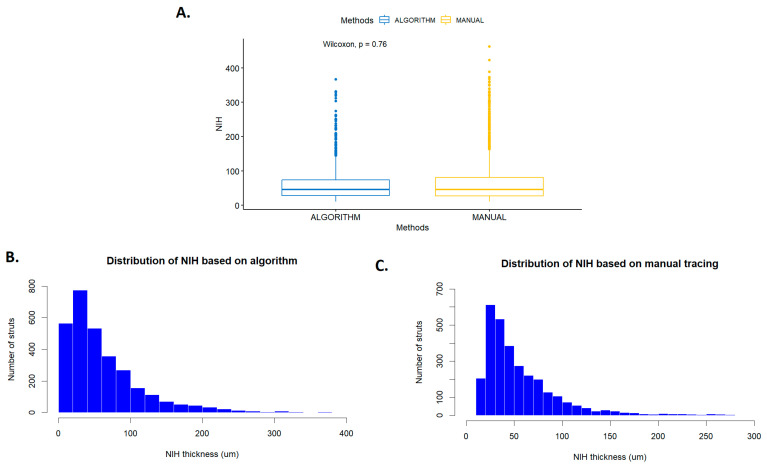
Box plots (**A**) and graphs (**B**,**C**) presenting results of neointimal thickness measurements obtained manually and by the algorithm.

The last step involved assessing neointimal thickness. A comparison of measurements obtained manually and by the algorithm is presented in Figure 5 using box plots. Both box plots show a similar right-skewed asymmetry and there was no statistically significant difference between these two methods (manual: median 44 (Q1:30–Q3:73) vs. algorithm: median 45 (Q1:27–Q3:83), *p* = 0.76). There are quite a few outlier measurements, but in some frames, the neointima measured by both methods was very thick. Also, manual tracing was characterized by greater variability in thickness < 100 μm in neointimal thickness distribution. These differences are likely due to the analyst’s ability to accurately determine where the vessel lumen begins while the algorithm includes the entire pixel. Additionally, the measurement of neointimal thickness highly depends on the detection quality of the lumen border close to the assessed strut.

## 4. Discussion

This study aimed to assess the algorithm’s efficacy for automated quantitative analysis of the vessel lumen and metallic strut tissue coverage with intravascular OCT images one month following coronary stent implantation. The algorithm has proven to be a reliable tool that facilitates and hastens OCT analysis, providing sustainable PPV and TPR in strut detection, distinguishing covered and uncovered struts, and estimating both lumen and stent areas. Moreover, measurements of neointimal thickness were convergent between the analyst and the algorithm.

Previous studies have confirmed that OCT is a reproducible and reliable method for strut coverage assessments, based on good intra- and interobserver agreement. However, it is worth emphasizing that all analysts in these studies were highly experienced, including CoreLab analysts and interventional cardiologists with a wide knowledge of OCT assessments [22,23,24,25]. Moreover, one of these studies showed that intraobserver and interobserver agreement highly depends on the zoom settings used during OCT evaluation of strut coverage. It differed less among CoreLab analysts than among interventional cardiologists familiar with OCT [24]. As mentioned before, OCT analysis of stent healing is exhausting. The time required for a single stent examination varies depending on image quality and the analyst’s experience. Another factor that may influence assessments of vascular healing is the sampling intervals. In clinical studies examining the safety and efficacy of different stent platforms, not all obtained OCT frames are analyzed. Usually, 1 mm sampling intervals are used, accelerating and facilitating image evaluation [26,27]. Unfortunately, this may interfere with outcomes. In patients treated for ACS, strut-level measurements of neointimal healing and strut apposition two months following PCI differed significantly between the conventional 1 mm and 0.6 mm sampling intervals [28]. Ideally, all available OCT frames should be analyzed to avoid sampling bias, which significantly prolongs image evaluation. Therefore, automation of OCT evaluation would permit wider use of this method in clinical settings.

A few algorithms have been proposed to facilitate the assessment of strut coverage. Ughi et al. presented a fully automated method of analysis of stent strut apposition and coverage, involving the detection and segmentation of stent struts using the intensity profiles of the A-lines [29]. The algorithm was validated using OCT images and histological data from rabbit iliac arteries after implantation of metallic stents. Automatic, manual, and histological measurements showed good agreement in coverage assessment. Furthermore, the authors recommended a final visual examination of struts assigned as uncovered to distinguish them from struts with thin tissue coverage [30]. Another algorithm for quantifying stent apposition and neointimal coverage in OCT images was introduced by Nam et al. It consisted of several steps, including preprocessing, detection of vessel lumen, then classification of struts into true and false based on their features, using an artificial neural network with one hidden layer and ten nodes. Finally, each strut’s protrusion distance and neointimal thickness were measured automatically. The proposed method demonstrated high TPR and PPV (above 90%) in detecting, classifying, and distinguishing between covered and uncovered struts [31]. Lu et al. presented fully automated machine learning-based software (OCTivat-Stent) for comprehensive stent analysis, which showed excellent agreement with manual measurements and provided a significant reduction in time required to perform analysis compared with fully manual evaluation of every stent frame with improved interobserver agreement [32]. Most recently, Young et al. proposed a deep learning algorithm for detecting stents with either thin (≤0.3 mm) or very thick tissue coverage (>0.3 mm). Also, this method provided analysis of the stent area for vessels with multiple layers of stents. The method was proven to be accurate with manual measurements [33]. Of course, even fully automated methods have some limitations. Usually, detection is disturbed by an overlying thrombus, thick neointimal tissue coverage, or a large amount of residual blood in the vessel lumen.

The presented algorithm also had some difficulties with the correct strut identification. In some cases, measurements were disturbed due to the presence of macrophages or calcifications. Ghost strut artifacts, i.e., a multiplied strut in the shadow area, can also disrupt detection. The algorithm will also fail when two struts are close to each other and when the strut or its shadow is not clearly visible in the image. However, even experienced analysts have difficulties during manual assessment in such cases. Another obstacle was determining the stent area in the frames where stent struts were visible in only a limited part of the vessel circumference (Figure 2). Also, it should be noted that the presented algorithm was characterized by high TPR in detecting uncovered struts, but the PPV was 77.3%. Comparing this with the results of the algorithm described by Nam et al. (PPV > 97%), it is low [31]. Ughi et al. reported a correlation coefficient of 0.97 for the strut coverage measurements [29]. The method described by Lu et al. demonstrated sensitivity/specificity of 94%/90% in detecting uncovered struts [34] and, in another study, 82%/99% [32]. However, algorithm performance depends significantly on case quality and difficulty, so we cannot compare these results directly. Moreover, in this study we used OCT images from earlier follow up, with numerous thinly covered struts. Also, the steps of the algorithm may be the cause of these results. Detected objects (strut shadows) are used to create a mask, and then smoothed, followed by a series of morphological operations. These steps may remove thin layers of neointima, making the struts appear uncovered.

On the contrary, automated algorithms reduce the labor and effort required to analyze the OCT examination. Errors are repeatable and can be easily caught, which may limit the number of analysts needed and reduce interobserver variability. The algorithms may be beneficial in trials regarding the thrombogenicity of new struts. Furthermore, automated assessment of arterial healing might allow safe DAPT discontinuation in clinical practice, which is an exciting and promising alternative. However, no guidelines on individualizing DAPT duration are grounded on OCT evaluation of arterial healing. In the DETECT-OCT study, investigators successfully tested early DAPT discontinuation based on the percentage of uncovered struts at three-month follow-ups. Patients with favorable vessel healing (<6% of uncovered struts) were assigned to three-month DAPT. A short OCT-based DAPT regimen was associated with a low incidence of composite events, demonstrating the feasibility of DAPT discontinuation depending on strut coverage [35]. These data are promising, but several limitations must be considered for OCT-tailored DAPT discontinuation. First, even if we automate neointimal coverage assessment, the cost of early OCT examination is high. Second, OCT imaging is an invasive procedure that can be associated with potential complications. Lastly, it must be stressed that delayed arterial healing is not the sole risk factor of ST. Nevertheless, in selected patients, OCT-tailored DAPT discontinuation may be clinically beneficial.

This study evaluated the proposed algorithm using only OCT images performed one month following stent implantation. It was confirmed that both stent platforms used in this study demonstrated favorable neointimal coverage at one month follow-up [26,27]. Therefore, this timepoint was chosen to test the algorithm, ensuring the presence of mostly covered struts, including struts with thin and thick tissue coverage. Furthermore, the duration of DAPT is still an intensely debated topic, and one month of DAPT is the shortest duration that was tested in clinical trials with novel drug-eluting stents (DES) and approved by the European Society of Cardiology in HBR patients [36]. Clinical trials demonstrated that, in this population, one-month DAPT combined with PCI with novel stent platforms is superior to PCI with bare metal stents (BMS) and one-month DAPT [37,38,39]. Further clinical trials regarding the duration of DAPT are clearly needed, as it is a complex issue.

## 5. Study Limitations

There are several limitations to this study. Firstly, we did not directly compare the presented algorithm with other similar software, but to our knowledge, these algorithms are not open-source available yet. Secondly, the algorithm was tested using OCT images one month following stent implantation; therefore, as for now, it cannot be applied to other clinical scenarios. Also, there is no available histological validation of the proposed method. Lastly, we included 24 pullbacks in this analysis, which might be insufficient and add bias to the results.

## 6. Conclusions

Automating OCT analysis of strut coverage might be beneficial in experimental and clinical settings. The proposed algorithm demonstrated good agreement with manual measurements. Further clinical studies with a more diverse and larger sample size are needed to confirm its reliability, but initial results are promising.

## Figures and Tables

**Table 1:** Results of manual and by the algorithm detection and quantification of struts. Algorithm validation was conducted using positive predictive value (PPV) and true positive rate (TPR) metrics.

	Expert	Algorithm	Expert vs. Algorithm	
			PPV (%)	TPR (%)
Total struts	5668	5763	89.7	91.4
Uncovered	1338	1719	77.3	99.1
Covered	4330	3467	87.0	80.0

## Data Availability

The raw data supporting the conclusions of this article will be made available by the authors on request.
